# Book Review: Food Safety: Emerging Issues, Technologies, and Systems

**DOI:** 10.3389/fmicb.2017.00987

**Published:** 2017-05-31

**Authors:** Mujahid Iqbal, Yanfei Tao, Zonghui Yuan

**Affiliations:** ^1^National Reference Laboratory of Veterinary Drug Residues (HZAU)/MOA Key Laboratory of Food Safety Evaluation, Huazhong Agricultural UniversityWuhan, China; ^2^MAO Laboratory for Risk Assessment of Quality and Safety of Livestock and Poultry Products, Huazhong Agricultural UniversityWuhan, China

**Keywords:** emergent bacterial pathogen, food microbiology, food pathogen detection, food safety education, molecular methods

Food safety: emerging issues, technologies, and systems is a food safety science book that proposes a structural approach to learning the basics of addressing and understanding some of the key intricate issues which have emerged around the food industry. The book offers the groundworks for real world understanding of safety rules and initiatives of food safety, how to deal with composite data and computer systems, handling issues of whole grain traceability, safety education, issues of processing and production compliance, and detection of food pathogens. The book encompasses recent scientific developments in the food industry, which have been explained by experts for the purpose of improving communication, awareness, and education of the same. This paper seeks to provide a review of this book with regards to its contents and the current community needs.

In essence, this book that is authoritative and attractively designed. The titles and chapters in the book are meaty but properly referenced and contain thorough information and a comprehensive introduction to every covered topic in the book. The content of the book is useful both for those looking to begin food science careers and to established specialists. Accordingly, this makes it to be an excellent book. Five out of the six chapters of the first section of this book were written by authors from the Arkansas University in Fayetteville. The approach to how these chapters are drafted and explained is international, though with an exception of the chapter on computer systems in traceability of whole grain in beef production systems since this one focuses on US practice exclusively. The section has other chapters which entails using numerical calculated modeling in biotracing, microbial cradle tracking by use of indicator organisms like in production of fresh produce, current, and probable future achievements in using the indicators of microbial process to address the *salmonella* chicken carcass contamination and using molecular approaches to trace foodborne pathogens.

The next section of the book has eight chapters which deals with distinct pathogens and contemplates the usual suspects which are foodborne illness brought about by *Staphylococcus aureus*, control of *Salmonella* in the United States and the consideration of antimicrobials as compared to antibiotics, the current perspectives on *Campylobacter* among others. There are three chapters written by Carol Philips, Jodie Score, Stephen Forsythe, and Katie Laird from various parts of Britain. These chapters cover *Clostridium difficile, Cronobacter, Arcobacter*, and *Aeromonas*.

In the last section of the book, the emphasis is on training and education. To begin with, the first three chapters which are about education on retail food safety, food safety in markets of farmers, and food handler education. In this light, food handler education comprises of education through the media and education of senior school pupils and this is written from the perspective of the United States. Even with this being the case, the value of the potential readers of this book elsewhere is not diminished since the problems addressed by the authors and the solutions tend to have a universal application and thus proves useful to every nation around the globe. The book provides a comprehensive and detailed review of approaches for ensuring perfect food handler practice through several approaches especially training. The conclusion of the book is an account of concerns that surround beef production in the United States and an account on teaching and training on food safety in Europe and the United Kingdom. This is a title and a chapter which completes itself with a wide-ranging index and offers the best explanation which can be comprehended by anyone who can read English.

Molecular methods of fingerprinting continue to develop at incredible speed as their cost fall more rapidly. In this regard, the challenge that evolution presents is as big as always and has to be dealt with since even the microbial adversaries are dynamically changing in an extremely unpredictable way. As such, it is paramount to ensure there is effective food handler training which will produce an impact required by microbiologists and sociologists.

The ever-evolving world of food safety science requires people to be masters in almost everything and this book offers the required knowledge to attain the same. The three sections of the book offer comprehensive knowledge on several developments in food safety traceability and tracking. In addition, the authors have offered a green light on new developments in training, food systems and food safety education, and new strategies to study foodborne pathogen ecology. Hence, the book is a good companion to individuals dealing with food safety from around the globe.

## Author contributions

MI identified the book and wrote the review. YT and ZY reviewed and edited the manuscript. All authors read and approved the final manuscript.

### Conflict of interest statement

The authors declare that the research was conducted in the absence of any commercial or financial relationships that could be construed as a potential conflict of interest.

